# Gait Biomechanics Across BMI Categories in Adults: A Cross-Sectional Study

**DOI:** 10.3390/healthcare14091119

**Published:** 2026-04-22

**Authors:** Carmen García-Gomariz, Sonia Andrés-Reig, María-José Chiva-Miralles, Roi Painceira-Villar, José-María Blasco

**Affiliations:** 1 Ankle and Foot Research Advances Group—GAITP, Department of Nursing, University of Valencia, Av. Menéndez y Pelayo 19, 46010 Valencia, Spain; carmen.garcia-gomariz@uv.es (C.G.-G.); maria.jose.chiva@uv.es (M.-J.C.-M.); 2 Department of Nursing, University of Valencia, Menéndez y Pelayo Av. 19, 46010 Valencia, Spain; soniaandresreig@gmail.com; 3 Group of Physiotherapy in the Ageing Process: Social and Health Care Strategies, Department of Physiotherapy, University of Valencia, Gascó Oliag 5, 46010 Valencia, Spain; jose.maria.blasco@uv.es; 4 Heqol Research Group, Department of Nursing and Physiotherapy, Campus de Ponferrada, Universidad de León, 24401 León, Spain; 5 Department of Physiotherapy, University of Valencia, Gascó Oliag 5, 46010 Valencia, Spain

**Keywords:** gait analysis, obesity, body mass index, spatiotemporal parameters, foot biomechanics, PODOSmart^®^

## Abstract

**Introduction**: Although gait alterations associated with excess body weight have been widely studied, most available evidence comes from laboratory-based analyses, which limit ecological validity and the translation of findings into clinical practice. This study addresses this gap by examining gait biomechanics across BMI categories using portable sensor-based insoles that allow gait assessment in real-world conditions. **Methods**: A cross-sectional study including 96 adults categorized as normal weight (NW), overweight (OW), or obese (OB) was conducted. Gait biomechanics were recorded using PODOSmart^®^ intelligent insoles, which capture spatiotemporal and angular parameters during natural walking. Foot health, quality of life and comorbildities were evaluated throught valeted questionnarires. Differences between groups were analyzed using ANOVA and chi-square tests. Age and sex, known to influence gait, were comparable across BMI groups and were considered in the interpretation of the results. **Results**: Overall, the participants in the OB group exhibited reduced stride length, gait speed, and swing time, increased double-support time, and greater pronation–supination and progression angles than OW and NW participants. Partial eta-squared values (η^2^p) were predominantly medium to large, reinforcing the robustness of these between-group differences (e.g., double-support time, *p* > 0.001; η^2^p = 0.19). Individuals with obesity reported poorer general and foot health and more difficulty finding suitable footwear. BMI was also significantly associated with hypertension, dyslipidemia, arthritis, and depression (all *p* <0.05), whereas diabetes, cardiopathies, knee pain, and fatigue andwalking or social activity limitations showed no significant differences. **Conclusions**: By using portable gait analysis technology in ecological conditions, this study provides novel evidence of clinically meaningful gait impairments across BMI groups. Higher BMI is associated with clinically relevant gait impairments, poorer perceptions of foot and general health, and a higher prevalence of several comorbidities.

## 1. Introduction

Human gait, understood as the mechanism of human movement, is a rhythmic process acquired in childhood and perfected over the years, resulting in individual, distinctive patterns [1]. Its execution emerges autonomously, thanks to complex neural networks and central pattern generators that coordinate locomotion [2]. The ability to walk is also a key indicator of health status and is strongly associated with quality of life [3,4].

Gait patterns can be modified by intrinsic and extrinsic factors, among which the weight is of clinical and epidemiological relevance [5]. The increasingly sedentary lifestyle, characterized by mechanized work, less daily physical activity, passive leisure, and a diet high in calories and low in nutritional content, has contributed to the rise in overweight and obesity [6]. The World Health Organization classifies body mass index (BMI) into four categories: underweight, normal weight (NW), overweight (OW), and obesity (OB) [7]. This latter condition, defined as an energy imbalance between calories consumed and calories expended, has reached alarming proportions in recent decades, tripling in incidence and surpassing underweight mortality rates. Currently, more than one billion people are obese [7], and data show a high prevalence of overweight and sedentary lifestyles in the adult population [8,9]. In addition to its association with multiple chronic diseases, obesity modifies body biomechanics and can alter the spatiotemporal, angular, and kinetic parameters of gait, increasing the risk of musculoskeletal and podiatric disorders [10], by increasing the mechanical load on the lower limbs and favoring the appearance of musculoskeletal and systemic comorbidities [11,12]. Individuals with obesity frequently present altered gait patterns, such as reduced stride length, slower gait speed, and prolonged double-support time, commonly interpreted as compensatory strategies to enhance stability under increased mechanical load [13]. These alterations include changes in agility, dynamic stability, and functionality, compromising daily mobility and favoring the appearance of associated pathologies [3,11,14,15,16]. The ongoing increase in obesity prevalence underscores the need for targeted analyses to better understand how excess weight influences gait patterns and podiatric and systemic health [6,17,18]. However, despite the volume of research available, many studies have been conducted in highly controlled laboratory settings, where walking conditions differ substantially from those of daily life. This reliance on laboratory environments limits the ecological validity of findings and may not accurately capture functional gait impairments experienced in real-world settings [19].Furthermore, the literature presents inconsistencies regarding which gait parameters are most affected by obesity, partly due to heterogeneous methodologies, small sample sizes, and variations in data capture technologies.

These limitations reveal a clear research gap: the need for studies assessing gait under natural, unconstrained conditions using validated portable systems capable of capturing biomechanical behavior in everyday environments. Such tools can provide more clinically meaningful and representative data and may detect alterations that traditional laboratory methods overlook.

In this context, portable sensor-based systems enable the quantification of gait in real-world environments, supporting the identification of gait alterations under real-world conditions [20,21]. It allows for the comparison of patterns between different BMI ranges and the exploration of their relationship with podiatric pathologies, systemic diseases, such as hypertension, high cholesterol, diabetes, arthritis, heart disease, circulatory disorders, or depressionand quality of life. Accordingly, the present study positions itself as a direct response to the methodological limitations identified in the existing literature. By employing PODOSmart^®^ portable insoles to collect gait data during natural walking, this work provides more ecologically valid evidence and advances current knowledge on how BMI influences gait biomechanics and related health factors.

Therefore, this study aimed to investigate whether there are differences in gait patterns between obese, overweight, and non-obese individuals, and to analyze the potential association with podiatric pathology, pain, systemic diseases, and overall health perception. To this end, gait at a self-selected comfortable speed was assessed using the PODOSmart^®^ system, a general health questionnaire, and the Foot Health Status Questionnaire (FHSQ) [22] were administered to evaluate the relationship among obesity, quality of life, and observed biomechanical alterations. The insoles were used to record 37 biomechanical parameters to identify gait differences across BMI categories

## 2. Materials and Methods

### 2.1. Design and Ethics

This cross-sectional observational study performed a biomechanical analysis of gait kinematic parameters in individuals aged 18 years and older, categorized by BMI. Gait parameters were assessed using the PodoSmart Insoles^®^ system (Digitsole SAS, Nancy, France) [21]. Participants were recruited from December 2023 to April 2024 at the municipal sports facilities in Enguera (Valencia, Spain) and at the Podiatry Clinic of the University of Valencia (Fundació Lluís Alcanyís, Valencia, Spain). The study design adhered to the scientific and ethical principles established in Helsinki, and the procedures were approved by the Ethics Committee of the University of Valencia (Spain) (no. 2528046/2023). All participants received verbal and written information about the study and provided written informed consent prior to participation

### 2.2. Participants

Recruitment was intentionally diversified across community settings (university clinic, municipal sports center, senior exercise program, and local community boards) to obtain a heterogeneous sample across age groups and activity levels, rather than a convenience sample.

The study population comprised 96 individuals aged 18 to 80 years, grouped according to WHO BMI criteria: Normal Weight (NW group, *n* = 30), Overweight (OW group, *n* = 31), and Obesity (OB group, *n* = 35). This sample was recruited at the Faculty of Nursing and Podiatry at the University of Valencia and from the “Enguera sé saludable” program, a municipally funded project for people over 60 years of age. Recruitment methods included WhatsApp, email, notice boards at the health center and the University Clinic, and an informational meeting. At this meeting, the study methodology was explained, and participants were encouraged to take part voluntarily. Those who signed the Informed Consent form were included. Those excluded were those with structural pathologies in the lower limbs that caused pain, those who used external aids for walking, such as canes, prostheses, or orthoses that prevented them from being measured by the sensors, patients with a shoe size less than 35, and those with a BMI less than 18.5. Participants with self-reported neurological disorders, vestibular dysfunctions, recent lower-limb injuries, or severe musculoskeletal conditions known to affect gait were not eligible for inclusion. Finally, a total of 96 subjects who met the inclusion and exclusion criteria participated out of 106 recruited. Participants from all BMI categories were drawn from both recruitment sources. Recruitment source did not differ systematically across BMI groups, and each group included individuals from the university clinic and the community program. Nevertheless, the use of multiple recruitment settings may introduce age-related heterogeneity, which is acknowledged and considered when interpreting the results and further discussed as a study limitation.

### 2.3. Procedures

The principal investigator (PI) verified compliance with the inclusion criteria and was responsible for managing the informed consent process. A podiatrist with over ten years of clinical experience assessed all participants, regardless of group assignment. The PI then recorded all measurements, anonymized the dataset by assigning a numerical code to each participant and prepare the database for analysis. Finally, the project biometrist independently processed and analyzed the anonymized raw data.

Data processing and statistical analyses were performed by a biometrist blinded to participant identity and group allocation using anonymized data; separation between data collection and analysis was maintained to reduce potential bias.

### 2.4. Measures

Demographic data and anthropometric measurements were obtained for all participants, including sex, age, height, weight, and shoe size. Gait characterization was performed using PodoSmart Insoles^®^ (Digitsole SAS, Nancy, France), software version 2.1.2, a portable system designed to quantify gait patterns under natural conditions. The device integrates wireless sensors into an insole compatible with most footwear, generating spatial, temporal, and kinematic indicators. The raw inertial sensor data are processed using predefined, validated proprietary algorithms that extract spatiotemporal and angular gait parameters [21].

All assessments were scheduled for the morning and at a uniform time (between 9:00 and 11:00 a.m.), with participants asked to arrive within this timeframe during the data collection period. For the recording, each subject walked for two minutes at a self-selected comfortable pace in an 18-m corridor. During this period, participants walked back and for along the corridor, performing turns at each end. The insoles were placed in the shoes according to the previously described protocol, and participants were instructed to wear their usual footwear to maximize comfort and avoid artificial modifications to their gait. All participants wore sports footwear during gait assessment; however, footwear models were not standardized beyond this general classification. Before the final recording, the research team provided verbal explanations and a practical demonstration of the procedure. Each participant repeated the test twice, and the average of the two values was used for the analysis. The PODOSmart^®^ system automatically excluded turning phases as well as acceleration and deceleration strides, retaining only steady-state straight-line walking strides for analysis. Across the two trials, an average of approximately 90–120 valid strides per participant were obtained, and gait parameters were averaged across these valid strides prior to statistical analysis.

Of the 37 biomechanical parameters generated by the PODOSmart^®^ system, only those previously validated in reliability and accuracy studies (ICC 0.75–0.99) were selected for analysis [20,23]. This decision ensured that the study focused exclusively on gait variables with demonstrated methodological robustness and clinical interpretability, as recommended by PODOSmart^®^ validation literature. Therefore, of all the gait parameters derived from the PodoSmart^®^ system, the following were selected for analysis: Stride length, Cadence (steps/minute), Speed (km/h), Contact time (ms), Swing time (ms), Double support time (ms), Support phase time (ms), Heel strike angle (deg), Toe-off progression angle (deg), Propulsion time, Step progression angle (deg), Stepping (deg), Toe-off angle (deg), and Circumduction (cm). The description of these variables and the previously reported ICC values is shown in Table 1. The ICC was categorized as follows: low reliability < 0.5; moderate reliability 0.51 to 0.75; high reliability 0.76 to 0.9; and excellent reliability > 0.9.

In addition to the biomechanical gait assessment, two complementary instruments were administered to all participants. First, the Foot Health Status Questionnaire (FHSQ), a validated tool that evaluates multiple domains related to foot health, pain, function, and foot-specific health-related quality of life, was completed. For the qualitative analysis, specific FHSQ items related to foot health, general health, and social aspects were selected. Second, participants responded to a general health questionnaire specifically designed for this study, which collected categorical information on chronic health conditions (e.g., hypertension, dyslipidemia, diabetes, cardiopathies, circulatory disorders) and several health-perception items (e.g., general health status, difficulty finding comfortable footwear, foot pain, fatigue while walking, or difficulty finding shoes). Both questionnaires consisted of nominal or ordinal response scales and were self-administered in a structured format prior to gait testing.

### 2.5. Data Analysis

Preliminary analyses confirmed no significant differences between groups in age or sex distribution, and all participants reported engaging in regular physical activity (≥2 days/week), reducing the influence of sedentary behavior as a confounder.

A descriptive statistical analysis was performed to summarize the sample’s characteristics using measures of central tendency (mean, minimum, maximum, etc.), measures of skewness, and dispersion tests (standard deviation). Data normality was assessed for each group independently using the Kolmogorov-Smirnov and Shapiro-Wilk tests to determine if the evaluated parameters followed a normal distribution. To compare gait parameters among the different BMI groups (NW, OW, and OB), one-way ANOVAs were performed between subjects, and an F-value was calculated to determine if there were significant differences both between and within groups using post-hoc tests (Tukey or Games-Howell). Although age, sex, and height are known to influence spatiotemporal gait parameters, particularly stride length and gait speed, adjusted analyses were not performed due to sample size considerations, and results were interpreted acknowledging the potential contribution of anthropometric differences. Effect sizes were calculated using partial eta-squared (η^2^p). Descriptors were applied as follows: negligible η^2^p < 0.01, small 0.01 ≤ η^2^p < 0.059, medium 0.06 ≤ η^2^p < 0.139, and large η^2^p ≥ 0.14. Categorical data obtained from the personal interview and from FHSQ items were analyzed using chi-square tests to assess the association between BMI categories and various health-related variables. For contingency tables with expected cell counts below five, categories were collapsed when appropriate or Fisher’s exact test was used instead of the chi-square test to ensure validity of the analyses. These included self-reported comorbidities, perceived general health, foot-related perceptions, and other nominal or ordinal responses.

An a priori power analysis confirmed that the final sample size (*n* = 96) was sufficient to detect between-group differences with adequate statistical power.

## 3. Results

**Figure 1:**
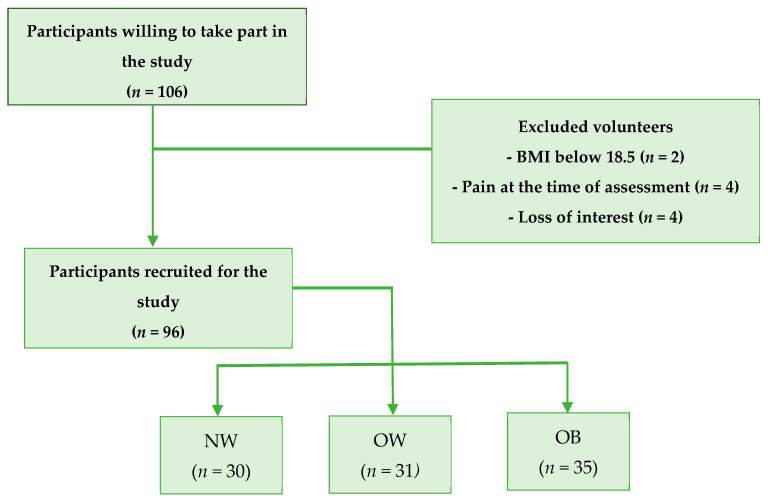
Flow chart of participants.

A total of 106 people were initially screened. After applying the inclusion criteria, 96 participants were eligible and included in the analysis (64.58% women and 35.42% men). Participants were classified into three groups: NW (*n* = 30), OW (*n* = 31), and OB (*n* = 35). The flowchart of the selection process is presented in Figure 1.

The demographic and anthropometric characteristics of the groups are shown in Table 2. As expected, significant differences were observed in weight, height, and BMI across groups, confirming the proper stratifications of participants. No significant between-group differences were found in age or baseline Foot Posture Index scores, reducing the likelihood that these variables influenced the gait outcomes.

The ANOVA revealed significant between-group differences in several spatiotemporal gait parameters. The most notable group effects were found for stride length in both limbs (right and left, *p* < 0.001); and double support time (*p* < 0.001). These variables are particularly relevant from a clinical perspective because reduced stride length and increased double-support time are well-established markers of gait inefficiency, balance compensation, and reduced functional capacity in adults with excess body weight.

Additional significant differences were observed in gait speed (*p* = 0.002), swing time (right: *p* = 0.001; left: *p* = 0.018), toe-off progression angle (right: *p* = 0.001; left: *p* = 0.004), circumduction angle of the left foot (*p* = 0.002), support phase time (right: *p* = 0.005; left: *p* = 0.013), propulsion time of the right foot (*p* = 0.021), right Step progression angle (0.008) and toe-off angle (*p* = 0.042). No significant differences were observed in the remaining gait parameters (Table 3).

Post hoc analyses demostrated that the OB group consistently differed from the NW and OW groups in all variables that reached statiscal significance. From a functional standpoint, these differences suggest that individuals with obesity adopt a more conservative gait strategy, characterized by shorter steps, prolonged support phases, and altered foot angles, to enhance stability and compensate for greater mechanical load on the lower limbs.

Effect size analyses revealed medium to large partial eta-squared (η^2^p) values in the parameters most affected by BMI.

The largest effects were observed for double support time (η^2^p = 0.19), right and left stride length (η^2^p = 0.18 and 0.17), right toe-off progression angle (η^2^p = 0.15), and left circumduction angle (η^2^p = 0.12). All η^2^p values are reported comprehensively in Table 3. These magnitudes indicate that the differences are not only statistically significant but also clinically meaningful, reinforcing the functional impact of excess body weight on gait biomechanics.

Chi-square analyses identified significant associations between BMI category and health-related variables (Table 4). The OB group showed a notably higher prevalence of hypertension (40%), hypercholesterolemia (42.9%), arthritis (28.6%), and depression (17.1%), as well as a greater frequency of poor general health, reduced foot health, moderate/severe foot pain, and difficulty finding suitable footwear. These findings align with the gait alterations observed, suggesting that biomechanical impairments may coexist with or contribute to broader musculoskeletal and systemic health burdens.

No significant associations were found for diabetes, cardiopathies, circulation problems, knee pain, or fatigue while walking; however, trends in some variables (e.g., higher fatigue prevalence in OB) indicate potential clinical relevance that may become significant in larger samples.

## 4. Discussion

This study analyzed the impact of body mass index on biomechanical gait parameters and whether obese individuals had a poorer quality of life, less favorable foot health, and a higher prevalence of associated diseases. Overall, the results confirmed that excess body weight significantly influences gait mechanics and various aspects of general and foot health.

First, the biomechanical analysis identified differences among the three groups. Specifically, these differences were observed in multiple spatiotemporal and angular variables, including bilateral stride length, speed, bilateral swing time and bilateral support phase time, double support time, right propulsion time, right and left toe-off progression angle, right step progression angle and right toe-off angle, and left circumduction. Effect sizes were predominantly medium to large, with the largest effects observed for stride length, swing time, speed, double support time, and toe-off progression angle, underscoring the strength of the between-group differences in these key gait parameters. Although variability was observed within groups, the medium-to-large effect sizes identified for key gait parameters, such as stride length and double-support time, suggest that the observed differences likely exceed typical measurement variability reported for these outcomes when assessed using validated wearable gait analysis systems.

These alterations are biomechanically consistent with the increased mass and inertia associated with higher BMI, which reduce forward propulsion efficiency, shorten step length, and prolong double-support as a compensatory strategy to enhance stability and reduce joint loading. Although excess body weight appears to play a central role in the observed gait alterations, these patterns should be interpreted within a multifactorial context, as age, stature, footwear characteristics, and comorbidity burden may also contribute to the biomechanical differences observed between BMI groups. This study is not novel simply by introducing new technology, but because it fills a clear methodological gap between laboratory-based gait research and real-world clinical biomechanics.

Pairwise comparisons suggested marked differences between the NW and OB groups. The OW group showed significant differences in fewer variables. This pattern may be explained by the fact that moderate increases in BMI do not yet produce sufficiently large mechanical constraints to disrupt gait efficiency; in some cases, individuals in the OW range may even display slightly higher gait speed or stride length than NW participants due to higher muscle mass or differing activity patterns. In contrast, the OB group (>30 kg/m^2^), lacking a defined upper limit, may exhibit greater body and functional variability, which could explain more pronounced differences compared to the NW group.

The findings of our study partially align with previous research, such as that by Lai et al. [23], who observed that obese individuals exhibit lower speed, shorter stride length, less propulsive force, and greater double support, patterns that align with ours. Ahsan M. et al. [3] also reported significant differences between the three BMI groups in various gait variables; however, in their study, the NW group had the highest speed and stride length, while in ours, the OW group showed higher values than the other two groups, although there were no differences between NW and OW, which were very similar. However, the cadence in this study did not show significant differences. Regarding clinical and quality of life variables, our qualitative analysis showed that people with obesity experience a greater burden of health problems. Foot pain was significantly more frequent in this group, consistent with the studies by Li J. et al. [10] and Dufour A. et al. [14].

Although knee pain did not show a significant association with BMI in our study, the overweight (OW) group reported the highest prevalence, followed by the obese (OB) group, indicating the need to explore this variable in larger samples. On the other hand, difficulty finding suitable footwear showed significant differences in the obese (OB) group, and no previous studies have specifically analyzed this variable. In terms of quality of life, we observed that obese individuals reported worse overall health, a finding consistent with results from another research [24]. The variable “fatigue while walking” did not reach statistical significance, although more than 30% of the obese group responded affirmatively, suggesting a possible effect that could emerge with a larger sample size. Other investigations [25] have associated increased body weight with a greater perception of fatigue during walking, which reinforces this interpretation. Given the number of health-related variables and questionnaire items analyzed, the following chi-square findings should be interpreted as exploratory, and the observed associations considered hypothesis-generating rather than confirmatory.

The association between BMI and chronic diseases was also evident: five of the eight variables studied (presence of global disease, hypertension, high cholesterol, arthritis, and depression) showed significant relationships. Our findings are consistent with previous studies linking obesity with hypertension [26], arthritis [10] and depression [27,28]. However, we did not observe significant associations with diabetes or heart disease, unlike studies such as those of Lai P. et al. and Houston D. et al. [24,29], which may be due to specific characteristics of our sample or variations in the diagnostic criteria used. Regarding cholesterol, our results align with studies such as the one from the University of Cambridge [30], which documented a higher prevalence of dyslipidemia in people with obesity. Taken together, these findings underscore clinically relevant implications: gait alterations associated with higher BMI may compromise functional mobility, increase fall risk, and accelerate lower-limb overloading, highlighting the importance of early biomechanical assessment. Moreover, these results may guide rehabilitation strategies aimed at improving propulsion, enhancing stability, and promoting efficient gait mechanics in individuals with excess body weight.

It is important to highlight the notable scarcity of studies that analyze the relationship between obesity and biomechanical gait parameters in such a comprehensive manner. Furthermore, many of the published studies have small sample sizes (28 participants in Li J. et al. [10]; 40 in Ahsan M. et al. [3]).

From a practical perspective, the present findings may help inform future exercise-based interventions aimed at improving functional gait patterns in individuals with overweight and obesity. Although the present study is descriptive, previous evidence suggests that structured physical exercise programs may contribute to improvements in gait efficiency and stability, and the gait alterations identified here may represent relevant targets for future intervention studies rather than direct clinical prescriptions [31].

Despite the robustness of the findings and the larger sample size compared to previous studies, this work is not without limitations. One of the main limitations is that, although the sample is larger than that typically used in similar research, it is neither large nor sufficiently representative to enable detailed stratification by age group or sex. Additionally, although recruitment was intentionally diversified across several community settings to increase heterogeneity, the non-probabilistic nature of the sampling strategy may still introduce selection bias. Therefore, the results should be interpreted with caution, as the representativeness of the sample may limit the generalizability of the findings to the broader adult population. Similarly, the number of participants did not allow for an in-depth analysis of the role of the Foot Posture Index (FPI); a larger sample size would have allowed for the classification of individuals into more specific subgroups (supinated, neutral, or pronated) and the examination of the influence of foot type on gait parameters and podiatric health. In addition, participants were recruited from different settings, including a university clinic and a municipally funded community exercise program for older adults. Although participants from all BMI categories were drawn from both recruitment sources, this recruitment strategy may have introduced age-related heterogeneity, which could have contributed to variability in gait parameters. Therefore, recruitment source and age distribution should be considered when interpreting the results.

Furthermore, as this is a cross-sectional study, it is not possible to establish causal relationships between BMI, gait biomechanics, and associated health problems. Although significant associations were identified, the temporal direction of these relationships cannot be determined, and longitudinal studies are required to clarify whether changes in BMI precede gait alterations or vice versa. Another limitation to consider is the use of self-reports to record some clinical and quality-of-life variables, which may introduce recall or perception biases, despite the use of validated questionnaires.

In addition, the lack of statistical control for certain potential confounding variables represents an important limitation. While preliminary analyses showed no significant differences between BMI groups in age or sex distribution, and all participants reported engaging in regular physical activity, other unmeasured factors, such as physical activity intensity, lower-limb strength, or long-term biomechanical adaptations, may have influenced gait parameters.

Furthermore, while PODOSmart^®^ insoles provide a reliable dynamic assessment, they do not allow the capture of certain three-dimensional kinetic and kinematic parameters that could complement the biomechanical interpretation. Future research should consider larger and more balanced samples by age and sex, integrate foot postural classification using the Foot Posture Index, and incorporate more complex instrumental methodologies or longitudinal designs to gain a deeper understanding of the mechanisms linking obesity to changes in gait, foot health, and associated diseases.

## 5. Conclusions

This study demonstrates that a higher BMI is strongly associated with significant alterations in gait biomechanics, poorer perceptions of overall and foot-related health, and a higher prevalence of several comorbidities. Obese individuals showed consistent impairments in key gait parameters, including stride length, gait speed, swing time, double support time, and flat-foot phase duration, with medium-to-large effect sizes (η^2^p ≈ 0.12–0.19; all *p* ≤ 0.020), highlighting the magnitude of the functional impact of excess body weight. Furthermore, the obese group was associated with poorer perceived overall health, poorer foot health, greater foot pain, and higher rates of hypertension, dyslipidemia, arthritis, and depression (all *p* < 0.005). Although no significant associations were found with diabetes, heart disease, or knee pain, the observed trends suggest that a larger sample size may reveal additional clinically relevant patterns.

From a practical perspective, these findings highlight the clinical value of gait analysis for identifying functional alterations associated with excess body weight. The identification of reduced stride length (*p* < 0.001), increased double-support time (*p* < 0.001), and altered propulsion patterns may assist clinicians in assessing balance, mobility efficiency, and lower-limb loading in individuals with overweight and obesity, while these results should be interpreted as descriptive given the cross-sectional design.

Taken together, these findings reinforce the importance of addressing obesity not only as a metabolic condition but also as a factor that compromises gait performance, musculoskeletal health, and overall well-being. Future research should seek to confirm these findings using longitudinal designs that allow for causal inference and the examination of temporal relationships between changes in BMI and gait biomechanics. In addition, studies with larger and more representative samples, improved control of confounding variables, and the integration of kinetic and three-dimensional gait analyses would provide a more comprehensive understanding of the mechanisms linking obesity to functional mobility impairments.

## Figures and Tables

**Table 1:** Measurements for gait analysis.

Name of Variable	Definition	ICC Value Reported
Stride length	Distance covered by the Right foot during one complete gait cycle	0.912
Cadence	Number of steps taken per minute.	0.990
Speed	The speed at which the body moves in a straight line while walking.	0.916
Contact time	Duration for wich of the foot remains in contact with the ground during a step.	0.989
Swing time	The duration between foot lift and heel strike for each foot during the stride cycle is used to determine the swing time, the length of the gait cycle when the limb is not in contact with the ground.	0.918
Double support	This is the percentage of the gait cycle during which both limbs contact the ground.	0.784
Support phase duration	The stance phase’s first sub-component. When the heel of the right foot strikes the ground and absorbs the impact, the support phase begins. It comes to an end when the toes touch the ground.	0.862
Heel strike angle	Angle at which the heel first contacts the ground during walking	0.930
Toe-off progression angle	Angle at which the toe lifts off the ground at the end of the stance phase	0.975
Propulsion phase duration	The stance phase’s third sub-component. The time between the heel and toe lifting events is referred to as propulsion.	0.799
Step progression angle	During the flat foot phase, the angle between the walking path and the foot’s orientation.	0.755
Stepping	Foot flexion/extension angke relative to the ground at initial contact.	0.914
Circumduction	Perpendicular to the progression line, the distance between the center of each foot’s heels.	0.949

ICC values are reported from prior PODOSmart^®^ validation studies and were not recalculated in the present sample.

**Table 2:** Anthropometric characteristics of the study population (*n* = 96).

	NW Group (*n* = 30) Mean (SD)	OW Group (*n* = 31) Mean (SD)	OB Group (*n* = 35) Mean (SD)	Between-Group Differences *p*-Value
Age (years)	44.76 (21.8)	53.7 (18.1)	57.0 (21.3)	0.053
Height (cm)	166.1 (10.2)	166.64 (8.2)	160.9 (7.3)	0.014 *
Weight (kg)	60.8 (7.1)	76.9 (7.1)	90.7 (16.7)	<0.001 *
Body mass index (kg/m^2^)	22.0 (1.7)	27.7 (1.42)	34.9 (4.3)	<0.001 *
FPI-R	2.2 (3.9))	2.4 (3.7)	2.8 (3.9)	0.804
FPI-L	2.3 (3.8)	2.9 (3.7)	2.8 (3.8)	0.779

* Statistically significant (*p* < 0.05).

**Table 3:** Between-group comparisons of gait parameters across BMI groups (one-way ANOVA with post-hoc tests).

Variable	NW Group (*n* = 30)	OW Group (*n* = 31)	OB Group (*n* = 35)	*p*-Value	Significant Pairwise Differences	η^2^p **
Stride length (m) R/L	1.45 ± 0.14/ 1.36 ± 0.10	1.48 ± 0.16/ 1.37 ± 0.11	1.31 ± 0.18/ 1.22 ± 0.17	<0.001 *	NW–OB (0.002); OW–OB (<0.001) NW–OB (0.001); OW–OB (<0.001)	0.18/0.17
Cadence (steps/min)	113.5 ± 8.37	113.19 ± 6.82	114.62 ± 9.39	0.766	–	–
Speed (km/h)	4.83 ± 0.56	4.90 ± 0.62	4.37 ± 0.75	0.020 *	NW–OB (0.018); OW–OB (0.040)	0.12
Contact time (ms) R/L	636.1 ± 60.6/ 607.3 ± 68.5	638.5 ± 45.3/ 618.5 ± 41.0	659.6 ± 73.4/ 641.5 ± 69.3	0.235/0.076	–	–
Swing time (ms) R/L	404.5 ± 29.7/ 394.4 ± 28.6	406.3 ± 21.1/ 391.3 ± 26.1	383.8 ± 29.1/ 375.0 ± 32.4	0.001 */0.018 *	NW–OB (0.007); OW–OB (<0.001)/ NW–OB (0.025)	0.13/0.08
Double support time (ms)	11.04 ± 1.46	11.55 ± 14.10	12.95 ± 2.25	<0.001 *	NW–OB (0.018); OW–OB (0.040)	0.19
Support phase time (ms) R/L	305.8 ± 50.5/ 290.1 ± 50.6	315.2 ± 48.4/ 303.9 ± 44.4	350.9 ± 65.6/ 332.3 ± 71.6	0.005 */0.013 *	NW–OB (0.010); OW–OB (0.045)/ NW–OB (0.011)	0.10/0.08
Heel strike angle (deg) R/L	−18.2 ± 4.2/ −17.0 ± 5.0	−17.9 ± 8.3/ −15.5 ± 8.0	−17.0 ± 3.7/ −15.4 ± 4.6	0.671/0.522	–	–
Toe-off progression angle (deg) R/L	−4.57 ± 4.37/ −7.50 ± 4.15	−1.70 ± 5.76/ −3.98 ± 4.80	−0.17 ± 2.67/ −5.42 ± 3.20	0.001 */0.004 *	NW–OB (0.009)/ NW–OB (<0.001)	0.15/0.11
Propulsion time (ms) R/L	228.9 ± 30.3/ 222.2 ± 34.7	223.7 ± 23.4/ 215.1 ± 21.2	208.9 ± 33.8/ 204.2 ± 32.5	0.021 */0.057	NW–OB (0.022)	0.07
Step progression angle (deg) R/L	8.66 ± 5.25/5.03 ± 4.98	11.48 ± 6.90/ 7.06 ± 6.25	13.20 ± 4.88/ 6.82 ± 5.23	0.008 */0.287	NW–OB (0.006)	0.09
Stepping (deg) R/L	26.66 ± 8.27/ 27.80 ± 8.73	26.90 ± 6.92/ 28.19 ± 6.95	23.80 ± 8.58/ 25.17 ± 8.50	0.215/0.259	–	–
Toe-off angle (deg) R/L	54.0 ± 8.44/ 51.1 ± 7.78	51.9 ± 4.47/ 49.4 ± 5.72	48.7 ± 10.7/ 47.9 ± 7.29	0.042 */0.186	NW–OB (0.035)	0.06
Circumduction (cm) R/L	3.90 ± 1.34/ 3.26 ± 1.38	3.41 ± 1.25/ 2.93 ± 0.77	3.31 ± 1.18/ 2.34 ± 0.83	0.152/0.002 *	-/NW–OB (0.007)	0.12

* Statistically significant (*p* < 0.05) ** Partial eta squared.

**Table 4:** Contingency tables and prevalence distributions of health-related variables across BMI groups.

	NW Group (*n* = 30) %	OW Group (*n* = 31) %	OB Group (*n* = 35) %	Chi-Square *p*-Value
Hypertension	10,3	25,8	40	0.027 *
Cholesterol	13.8	25.8	42.9	0.034 *
Arthritis	3.4	12.9	28.6	0.020 *
Depression	0	6.5	17.1	0.043 *
Diabetes	0	6.5	8.6	0.291
Cardiopathies	3.4	0	8.6	0.217
Circulation problems	0	6.5	2.9	0.358
Fatigue while walking	17.2	12.9	31.4	0.153
Social activity	34.5	30	52.9	0.335
Knee pain	17.2	35.5	28.6	0.28
Foot pain (moderate/severe)	10.3	16.2	28.6	0.049 *
Difficulty finding shoes	24.1	29	48.6	0.029 *
Foot health (regular/poor)	13.4	29	48.6	0.009 *
General health (poor)	0	0	14.3	0.001 *

* Statistically significant (*p* < 0.05).

## Data Availability

The data presented in this study are available on reasonable request from the corresponding author. The data is not publicly available due to privacy and ethical restrictions.

## Abbreviations

The following abbreviations are used in this manuscript:

BMI	Body Mass Index
NW	Normal Weight
OW	Overweight
OB	Obese
FHSQ	Foot Health Status Questionnaire
